# Combined Enucleation and Avulsion of a Fibrous Tumor of the Uterus

**Published:** 1871

**Authors:** J. Matthews Duncan

**Affiliations:** Lecturer on Midwifery, etc.


Article II.—*Combined Enucleation and Avulsion of a Fibrous Tumor of the Uterus.* By J. Matthews Duncan, M.D., Lecturer on Midwifery, etc.

---

I put on record the following case because it is a remarkable example of an operation which is not frequently performed. This procedure, which I have now many times resorted to, and always with success, consists in removing a uterine fibroid from its bed of muscular tissue by seizing and pulling by strong volsellæ. I have resorted to it only in cases in which the discharges—bloody, serous and purulent—from the tumor were so large and so long continued as to imperil the patient's life; and I have only resorted to it when no other means of relief seemed available. I proceed not to enucleate the tumor or separate it from its attachments, and then to remove it, but simultaneously to remove and enucleate, or to enucleate by avulsion. The operation is long and laborious, and has no claim to elegance or brilliancy, being rather rude and coarse. There are, no doubt, many dangers in the operation which I have been fortunate enough to avoid, some of which I have described in former papers* in this journal, but which I am not prepared here to enter upon.

The great difficulty of the operation is merely mechanical. It does not lie in the enucleation: some tumors are connected with the uterus by tissues so delicate as to offer scarcely any resistance to the disembedding force: others are united at points by strong bands of muscular and connective tissue to their beds, and these bands seem to me to be connected chiefly with the part of the tumor which is last separated; and in the operation it may be difficult to distinguish such connecting bands from a prolongation or continuation of the tumor itself. The former loosely-connected tumours come away easily, if small, and are as smooth and clean and rounded as an egg or a pear. The latter are much lacerated, especially if large, and have numerous tags and bands attached, of various dimensions and lengths.

The great difficulty of the operation is in pulling the tumour through the undilated passages. The operation, indeed, has some analogies with the obstetric forceps operation, and, like it, should not be done quickly or hurriedly. The first obstruction is in the

---

* See especially this journal for January and February, 1867, and for December, 1869.

cervix uteri, the opening also in the wall within which the fibroid is lying, and its adhesions to its bed. These three sources of difficulty combined form the first cause of obstruction. The second, and in the case of a large tumor, the more powerful and embarrassing, is the perineum and orifice of the vagina, if these parts are in a natural or nearly natural state.

Mrs. S., æt. forty-three, had been twenty years married, sterile, enjoyed good health till somewhat above two years before the operation to be described. Then the menstrual flow became profuse, and were accompanied by pain. During the last year the bloody flux was constant and often profuse. She was under the care of Dr. Meikle, of Douglas, and had also other advice. Summoned by Dr. Meikle, I visited this patient on the 15th June, and removed a fibrous tumor weighing one pound and three-quarters. In the operation I was greatly aided by Dr. Meikle and his son.

The patient was extremely anæmic and weak, and had a bloody discharge. In the hypogastric region was a hard, rounded tumor, whose upper margin was on a level with the navel. Examination per vaginam discovered that the tumor lay high up; the cervix, dilated to fully an inch in diameter, could just be well reached by the examining finger. The os had thin rigid lips. The tumor pressed on the cervix.

To enlarge the os, I cut its margin freely on each side with scissors. I then seized the tumor by a volsella, then by two, and kept pulling away, with few intermissions, except when the instruments tore their way out of the tumor. After about an hour's work, and repeated pushing back of the uterine wall by the finger, the tumor began to distend the perineum. But then further progress appeared unattainable. Continued efforts, however, brought it down a little farther, so as to be within reach of the knife; and I attempted to cut it spirally, but really only succeeded in making an oblique cut into it, which allowed the lower portion to come through the orifice of the vulva. To this part a strong cord was now applied for the purpose of increasing traction. At length, after the whole operation had lasted about two hours, and after some pieces had been torn away, the whole tumor was extracted; but before its separation was complete, two thick bands had to be cut through.

The tumor was everywhere raw or recently denuded, and was quite bare of capsule or any covering. Some hæmorrhage took place during the operation, and continued for some hours after it.

The woman was, after the operation, in a state of the greatest exhaustion, and occasionally very faint and pulseless. When I left her, in charge of Dr. Meikle, junior, I had the gravest apprehensions as to her rallying. But she made a slow and uninterrupted recovery. She menstruated naturally in July.—*Edinburg Medical Journal.*

